# Burnout syndrome and healthy lifestyle among Egyptian physicians: A cross-sectional study

**DOI:** 10.1371/journal.pone.0320146

**Published:** 2025-04-18

**Authors:** Nehal Mohamed Eisa, Mohamed A. M. El-Tabakh, Nourhan M. Kamal, Sara M. Gharbia, Mahmoud M. Samir, Wajid Syed, Mahmood Basil A. Al-Rawi, Ahmed Essam Abou Warda, Abdelrahman S. H. Refaee

**Affiliations:** 1 Clinical Research Department at Giza Health Affairs Directorate, MOHP, Giza, Egypt; 2 Department of Clinical Pharmacy, Faculty of Pharmacy, October 6 University, Giza, Egypt; 3 Department of Zoology, Al-Azhar University, Cairo, Egypt; 4 ICH-CRC, MOHP, Giza, Egypt; 5 Department of Clinical Pharmacy, College of Pharmacy, King Saud University, Riyadh, Saudi Arabia; 6 Department of Optometry, College of Applied Medical Sciences, King Saud University, Riyadh, Saudi Arabia; 7 Department of Pharmacotherapy, Faculty of Pharmacy, Florida University, Gainesville, Florida, United States of America; The World Bank Group, CANADA

## Abstract

**Introduction:**

The phenomenon of burnout and the lifestyle of physicians significantly influence the delivery of healthcare. Over time, burnout intensifies, negatively impacting professional performance, which in turn leads to decreased quality of treatment, patient satisfaction, and productivity. Additionally, it increases the occurrence of medical mistakes and turnover among physicians. In addition to the direct influence of lifestyle on those components.

**Aim of the study:**

The purpose of this study is to assess burnout syndrome among Egyptian physicians, as well as to investigate factors that contribute to burnout, especially demographic characteristics, lifestyle patterns, and health habits.

**Methods:**

A cross-sectional study examined burnout prevalence and determinants among 502 Egyptian physicians in different governorates. An electronic questionnaire was used to collect data for the study. Questionnaire covered socio-demographics, The abbreviated Maslach Burnout Inventory (aMBI), and The Health Lifestyle and Personal Control Questionnaire (HLPCQ).

**Results:**

Younger physicians under 30 showed higher burnout on emotional exhaustion and depersonalization scales, with significant findings (P = 0.047), (P <  0.01) respectively. Male physicians showed stronger depersonalization than females (P <  0.01). Burnout was higher among residents and fellowship trainees, with significant differences in depersonalization (P = 0.021). PhDs showed decreased burnout with significant outcomes (P = 0.002). Longer-working doctors had increased burnout in depersonalization (P = 0.005). Single doctors were more depersonalized than married ones (P = 0.025). Depersonalization was higher in childless people (P = 0.002). However, non-chronic illness physicians were more emotionally exhausted (P = 0.042).

**Conclusion:**

These findings highlight the intricate relationship between burnout and lifestyle among physicians. A healthy lifestyle, including diet, routines, social support, and physical activity was linked to reduced burnout, while dietary harm avoidance was negatively correlated. This suggests opportunities to enhance the well-being of medical professionals through lifestyle interventions.

## Introduction

The health and well-being of healthcare providers are essential for delivering high-quality patient care. Healthcare provider burnout is an unintended consequence of chronic workplace stressors, which can be emotional or interpersonal [1].

Burnout syndrome has emerged as one of today’s most serious psychosocial occupational disorders, resulting in significant costs for both healthcare providers and organizations [2]. Burnout is a syndrome of serious emotional exhaustion with poor work adaptation due to prolonged occupational stress [3]. Christina Maslach described it in terms of emotional exhaustion (EE), depersonalization (DP), and personal accomplishment (PA) [4]. EE manifests in feelings and sensations of being exhausted by the psychological efforts made at work, while DP is a response of detachment, indifference, and unconcern toward the work being performed and/or the people who receive it. PA is reflected in a negative professional self-evaluation and doubts about the ability to perform the job effectively [5].

Burnout syndrome has many negative effects on both the individuals who suffer from it and the organizations where these providers work. These effects are initially psychological in nature, but they later affect physical, biological, and behavioral health, which will have unfavorable organizational consequences [6]. Burnout can affect patient care by leading to poor care quality, medical errors, longer recovery times, and lower patient satisfaction [4]. There is an increased need to construct a plan for action to restore healthcare provider well-being: aligning personal and organizational values and allowing providers to practice different self-care strategies [1].

Previous studies concerning burnout among healthcare providers investigated a wide range of burnout predictors. These included demographic factors, professional and clinical practice characteristics, and workplace factors such as workload, work/life balance, job autonomy, and leadership issues. Mental health factors such as anxiety, as well as physical health factors, lifestyle patterns and habits, and psychosocial variables, may all increase the risk of burnout [1].

Lifestyle is an important factor that can influence healthcare providers’ burnout. For stress management, there is a need to improve health by empowering people to take control over their lives through daily health-related lifestyle choices [7]. A healthy lifestyle is significantly correlated with maintaining health and preventing disease [8]. According to WHO guidelines, a healthy lifestyle can lower the risk of preventable health problems and improve quality of life (QoL) [9]. Also, the occurrence of the COVID-19 pandemic exacerbated healthcare providers’ burnout, adding several physical and emotional stressors on frontline healthcare providers [10].

In order to effectively avoid or reduce the complex phenomena of burnout, this study intends to contribute to the existing literature on the subject by evaluating burnout syndrome among Egyptian physicians. The study will take into account a wider variety of factors that are known to predict and cause burnout.

## Subjects and methods

### Study design and data collection

A cross-sectional, descriptive observational study was designed to assess the prevalence and factors related to burnout among Egyptian physicians working in all health facilities in different governorates in Egypt. The study population includes Egyptian physicians of both sexes who were between 25-80 years old and exclude who were above 80 years old.

The sample size was calculated to be 390 subjects, we evaluated the present study on 502 participants taking into consideration that the total number of Egyptian physicians (governmental and private physicians) is 98,000, provided by the Central Agency for Public Mobilization and Statistics [11], the prevalence of burnout syndrome in Egypt is 36.4% [12], the power was 80%, the statistical level of significance was 0.05, and 10% expected non-response. The sample size was calculated using the Open Epi, Version 3, open-source calculator.

The study was conducted over six months, starting in January 2023. The study included physicians from 10 governorates out of a total of 27 governorates. The physicians were selected using non-probability sampling techniques, specifically convenience sampling, to capture a diverse range of participants across different regions.

Physicians were invited to participate through electronic communications via social media platforms, including WhatsApp and Facebook medical groups, as well as private accounts. The data was collected using a pre-designed, electronic, self-administered questionnaire, which was distributed through Google Forms.

### Measures of assessment

The study utilized three questionnaires: (a) A socio-demographic, occupational, and health-related characteristics questionnaire (16 questions). (b) The abbreviated Maslach Burnout Inventory (aMBI) (9 questions) [13]. (c) The Healthy Lifestyle and Personal Control Questionnaire (HLPCQ) (26 questions) [7].

The questionnaires were most likely distributed in the Arabic language to ensure clarity and understanding among participants. The translation process was carefully conducted following established procedures. Initially, the questionnaires were translated from English to Arabic by two independent bilingual experts. The translations were then compared and synthesized into a single version. A back-translation to English was performed by a separate bilingual expert to ensure the accuracy and equivalence of the content. Any discrepancies were resolved through discussion among the translators to achieve a final version.

In addition, an official approval from MindGarden was obtained to allow the use of the abbreviated Maslach Burnout Inventory (aMBI). This approval is mandatory for the usage of the questionnaire and was secured before the study commenced to comply with publication requirements.

The aMBI is a nine-item scale used for assessing burnout. It has three subscales: emotional exhaustion (EE), depersonalization (DP), and personal accomplishment (PA). Each subscale is assessed by three items. For each item, there is a seven-point Likert scale that ranges from never (0) to every day (6). The score for each item was summed up for each doctor. For emotional exhaustion and depersonalization, a higher score means greater burnout; this is the inverse for personal accomplishment. The score of each subscale could range from a minimum of 0 to a maximum of 18. A high score of EE and DP and a lower score of PA indicate a higher level of burnout [13]. The aMBI defined burnout as the presence of two or more ‘moderate’ scores: EE ≥  7, DP ≥  4, or PA ≤  14. The scores of each subscale are reported separately and cannot be added up as a total score.

The Healthy Lifestyle and Personal Control Questionnaire (HLPCQ): The HLPCQ consists of 26 items. It asks individuals to rate their lifestyle habits on a Likert-type scale (1 =  never or rarely, 2 =  sometimes, 3 =  frequently, and 4 =  always). There are 12 items about diet, 8 about daily time management, 2 about organized physical exercise, and 4 about social support and positive thinking. The reliability and internal consistency of the HLPCQ were satisfactory [7].

### Ethical considerations

Ethical approval for the study was obtained from the “Research Ethics Committee” in the Central Directorate for Research and Health Development in MOHP (REC No. 19-2022/22). The study was conducted in accordance with the ethical guidelines and principles outlined by the Declaration of Helsinki and the World Health Organization. Participants’ informed consent was obtained prior to their participation in the survey. The consent process included a clear explanation of the study’s purpose, procedures, potential risks, benefits, and the voluntary nature of participation. Participants were assured of the confidentiality and anonymity of their responses. Consent was actively obtained by requiring participants to indicate their agreement by clicking a consent button before proceeding to the survey.

### Statistical methods

Descriptive statistics, including means, standard deviations (SD), minimums, maximums, absolute frequencies, and percentages, were used to summarize the data. Chi-square (χ²) tests were performed using MiniTab V14 to determine whether the actual burnout survey response rates matched the expected rates. Principal component analysis (PCA) was employed to extract the elements of the Healthy Lifestyle and Personal Control Questionnaire (HLPCQ). Bartlett’s test was conducted to assess the adequacy of correlations between items, and a determinant value was calculated to check for multicollinearity (with the determinant close to zero indicating potential multicollinearity). The Kaiser-Meyer-Olkin (KMO) measure was used to evaluate the sample size adequacy. The correct number of derived components was determined using the scree plot method (identifying inflexion points) and Kaiser’s criterion of eigenvalues greater than 1. An orthogonal varimax rotation was applied to maximize the loadings of each item on the resulting factors, with items having loadings greater than 0.3 being considered significant for further analysis. Cronbach’s alpha values were calculated to assess the internal reliability of the variables. Following this, a multiple linear regression analysis was conducted to examine the relationship between burnout (as measured by the abbreviated Maslach Burnout Inventory) and the various predictors, including demographic data, HLPCQ scores, and other relevant variables. The regression model was adjusted for potential confounding factors to provide a more precise understanding of the determinants of burnout. The results from the regression analysis, including B values, p-values, and 95% confidence intervals, were reported to highlight the strength and significance of these relationships. Pearson’s rho correlation coefficient was also used for between-group comparisons where appropriate. Statistical significance was set at p <  0.05, and all analyses were conducted using SPSS version 28 for Windows.

## Results

### Demographic structure and employment categorization of participants

**Fig 1:**
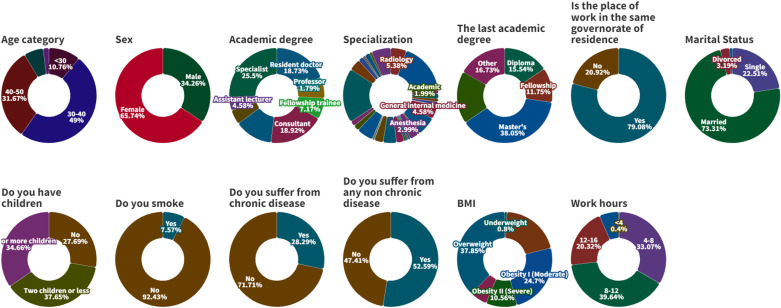
Donut chart shows Demographic structure and Employment categorization.

Five hundred and two Egyptian physicians participated in this study who were mostly (49%) in the age group (30-40) years with more female participants (65.73%); specialists compose 25.4% and significant participation of rheumatologists (18.52%). Most participants (38.04%) were holding a master’s degree regarding their level of education and also worked in the same governorate of residence (79.08%) for (8-12) hours (39.6%). The majority of participants were married (73.3%). Most of the participants weren’t smokers (92.4%) and didn’t suffer from chronic disease (71.7%) but were also overweight (37.84%). There was no significant difference in family size regarding to children’s number and suffering from non-chronic disease. (Table 1 and Fig 1).

**Table 1:** Demographic structure and Employment categorization.

Demographic structure	Category	Observed n (%)	*X* ^*2*^	P-value
Age category	<30	54 (10.75)	393.438	**<0.001**
	30-40	246 (49)		
	40-50	159 (31.67)		
	50-60	33 (6.57)		
	>60	10 (1.99)		
Sex	Male	172 (34.26)	49.7291	**<0.001**
	Female	330 (65.73)		
Academic degree	Resident doctor	94 (18.72)	246.147	**<0.001**
	Professor	9 (1.79)		
	Lecturer	27 (5.37)		
	Fellowship trainee	36 (7.17)		
	Consultant	95 (18.92)		
	Assistant specialist	65 (12.94)		
	Assistant Professor	25 (4.98)		
	Assistant lecturer	23 (4.58)		
	Specialist	128 (25.49)		
(Specialization	Radiology	27 (5.37)	901.486	**<0.001**
	Pediatrics	79 (15.73)		
	Academic	10 (1.99)		
	Pulmonology	6 (1.19)		
	Cardiology	9 (1.79)		
	Nephrology	5 (0.99)		
	Infectious diseases	6 (1.19)		
	Pathology	1 (0.19)		
	General internal medicine	23 (4.58)		
	Medical analysis	33 (6.57)		
	Audiology and Speech	3 (0.59)		
	Anesthesia	15 (2.98)		
	Nutrition	3 (0.59)		
	Otolaryngology	2 (0.39)		
	Oncological surgery	3 (0.59)		
	Plastic surgery and burns	3 (0.59)		
	General surgery	13 (2.58)		
	Orthopedic surgery	23 (4.58)		
	Ophthalmology	15 (2.98)		
	Cardiothoracic Surgery	1 (0.19)		
	Neurosurgery	2 (0.39)		
	Urological Surgery	1 (0.19)		
	Obstetrics and gynecology surgery	28 (5.57)		
	Oral and maxillofacial surgery	10 (1.99)		
	Dermatology and Venereology	9 (1.79)		
	Rheumatology	93 (18.52)		
	Family medicine	20 (3.98)		
	General medicine	9 (1.79)		
	Emergency	2 (0.39)		
	Oncology	2 (0.39)		
	Intensive care	9 (1.79)		
	Gastroenterology & Hepatology	4 (0.79)		
	psychoneurological	33 (6.57)		
The last academic degree	Diploma	78 (15.53)	107.582	**<0.001**
	Fellowship	59 (11.75)		
	Master’s	191 (38.04)		
	Ph.D.	90 (17.92)		
	Other	84 (16.73)		
Is the place of work in the same governorate of residence	Yes	397 (79.08)	169.849	**<0.001**
	No	105 (20.91)		
Marital Status	Single	113 (22.5)	681.06	**<0.001**
	Married	368 (73.3)		
	Divorced	16 (3.18)		
	Widower	5 (0.99)		
Do you have children	No	139 (27.68)	7.86853	0.02
	Two children or less	189 (37.64)		
	Three or more children	174 (34.66)		
Do you smoke	Yes	38 (7.56)	361.506	**<0.001**
	No	466 (92.4)		
Do you suffer from chronic disease	Yes	142 (28.28)	94.6693	**<0.001**
	No	360 (71.71)		
Do you suffer from any non-chronic disease	Yes	264 (52.58)	1.31661	0.246
	No	238 (47.41)		
BMI	Underweight	4 (0.79)	281.418	**<0.001**
	Normal weight	102 (20.31)		
	Obesity I (Moderate)	124 (24.7)		
	Obesity II (Severe)	53 (10.55)		
	Obesity III (Very severe or morbid)	29 (5.77)		
	Overweight	190 (37.84)		
Work hours	<4	2 (0.39)	281.406	**<0.001**
	4-8	166 (33.06)		
	8-12	199 (39.64)		
	12-16	102 (20.31)		
	>16	33 (6.57)		

**Table 2 and Figs 2**, **3, and 4** demonstrate the prevalence of low, moderate, and high burnout across various demographic factors and lifestyle characteristics among 502 Egyptian physicians. Notably, higher burnout was observed in younger physicians below the age of 30 on the emotional exhaustion (EE) and depersonalization (DP) scales, with significant findings (P = 0.047), (P <  0.01) respectively. Burnout levels declined with advancing age categories. Male physicians exhibited significantly higher depersonalization than females (P <  0.01). Residents and fellowship trainees showed a greater tendency toward burnout, with significant differences in depersonalization (P = 0.021) while in emotional exhaustion had higher burnout (P = 0.082). Burnout levels did not vary substantially across different specialties, as indicated by non-significant findings (P = 0.84). However, those with higher degrees, such as PhDs, had lower burnout levels with significant results (P = 0.002). But in emotional exhaustion showed lower exhaustion than other degrees (P = 0.363). Physicians working longer hours reported significantly higher burnout, depersonalization being notably elevated while decreased in emotional exhaustion (P = 0.076). Single physicians experienced more depersonalization compared to their married counterparts (P = 0.025). Additionally, those without children showed significantly higher depersonalization (P = 0.002). In contrast, in emotional exhaustion, married physicians had lower exhaustion than single ones (P = 0.061). Having children was associated with lower emotional exhaustion (P = 0.197). Chronic disease, smoking, and BMI did not significantly impact burnout prevalence, as indicated by non-significant findings (P = 0.892), (P = 0.711), (P = 0.773) respectively. However, physicians with non-chronic illnesses exhibited higher emotional exhaustion (P = 0.042).

**Table 2:** Burn out assessment throughout demographic structure of the participant.

Demographic structure	Levels	Personal Accomplishment			*X* ^2^	P-value	Depersonalization			*X* ^2^	P-value	Emotional Exhaustion			*X* ^2^	P-value
		*lb*	*mb*	*hb*			*lb*	*mb*	*hb*			*lb*	*mb*	*hb*		
Age category	<30	12	16	26	30.350	**<.001**	11	15	28	28.528	**<.001**	1	10	43	15.695	**0.047**
	30-40	101	54	91			101	60	85			20	39	187		
	40-50	95	25	39			73	47	39			13	21	125		
	50-60	17	7	9			22	6	5			8	2	23		
	>60	7	2	1			6	3	1			1	2	7		
Sex	Male	89	33	50	3.292	0.193	54	40	78	24.32	**<.001**	13	21	138	1.875	0.392
	Female	143	71	116			159	91	80			30	53	247		
Academic degree	Resident doctor	27	27	40	31.073	**0.005**	32	29	33	26.733	**0.021**	7	14	73	21.850	0.082
	Professor	6	1	2			7	2	0			4	1	4		
	Lecturer	15	6	6			13	5	9			1	3	23		
	Fellowship trainee	11	11	14			8	8	20			0	6	30		
	Consultant	58	13	24			40	27	28			8	11	76		
	Assistant specialist	26	12	27			24	22	19			7	11	47		
	Assistant lecturer	26	9	13			24	10	14			4	9	35		
	Specialist	63	25	40			65	28	35			12	19	97		
Specialization	Psychology and Neuroscience	15	8	10	52.808	0.84	15	8	10	79.238	0.095	3	6	24	55.985	0.752
	Hepatology and Gastroenterology	2	1	1			3	1	0			0	1	3		
	Intensive care	3	4	2			3	1	5			1	1	7		
	Oncology	2	0	0			1	1	0			0	0	2		
	Emergency Medicine	1	0	1			1	1	0			0	1	1		
	General Practitioner	3	1	5			5	1	3			1	2	6		
	Family Medicine	12	2	6			9	4	7			0	2	18		
	Rheumatology and Rehabilitation	42	20	31			56	22	15			12	24	57		
	Dermatology and Venereology	4	2	3			2	5	2			1	2	6		
	Oral and Maxillofacial Surgery	4	2	4			5	3	2			2	1	7		
	Obstetrics and Gynecology	16	5	7			9	5	14			4	3	21		
	Urology	1	0	0			0	0	1			0	0	1		
	Neurosurgery	1	0	1			1	0	1			0	0	2		
	Cardiac and Thoracic Surgery	0	1	0			0	0	1			0	0	1		
	Ophthalmology	6	4	5			5	5	5			1	3	11		
	Orthopedic Surgery	14	5	4			6	5	12			3	3	17		
	General Surgery	7	3	3			3	5	5			1	2	10		
	Plastic and Burns Surgery	1	1	1			2	0	1			0	0	3		
	Oncology	2	0	1			1	2	0			0	0	3		
	Otorhinolaryngology	1	0	1			0	1	1			0	1	1		
	Nutrition	2	1	0			2	0	1			0	0	3		
	Anesthesiology	11	2	2			7	5	3			2	2	11		
	Audiology and Speech	1	0	2			1	0	2			1	0	2		
	Medical analysis	14	7	12			19	9	5			3	7	23		
	Internal Medicine	11	6	6			10	6	7			1	2	20		
	Pathology	0	1	0			1	0	0			0	1	0		
	Infectious Diseases	2	1	3			1	4	1			1	0	5		
	Nephrology	1	3	1			2	1	2			0	1	4		
	Cardiology	6	1	2			5	2	2			0	1	8		
	Pulmonology	3	1	2			3	0	3			0	0	6		
	Academic	5	1	4			2	4	4			0	0	10		
	Pediatrics	33	13	33			26	24	29			4	5	70		
	Radiology	6	8	13			7	6	14			2	3	22		
The last academic degree	diploma	35	18	25	23.976	**0.002**	37	19	22	11.705	0.165	7	17	54	8.761	0.363
	fellowship	27	10	22			29	10	20			4	11	44		
	Master’s	88	38	65			83	51	57			18	27	146		
	Ph.D.	58	14	18			40	26	24			10	9	71		
	other	24	24	36			24	25	35			4	10	70		
Is the place of work in the same governorate of residence	Yes	193	76	128	5.025	0.081	173	104	120	1.534	0.464	34	57	306	.226	0.893
	No	39	28	38			40	27	38			9	17	79		
Work Hours	<4	2	0	0	15.259	**0.054**	1	1	0	21.833	**0.005**	0	1	1	14.211	0.076
	4-8	67	41	58			84	36	46			18	33	115		
	8-12	104	34	61			87	57	55			18	28	153		
	12-16	51	19	32			35	29	38			5	8	89		
	>16	8	10	15			6	8	19			2	4	27		
Marital Status	Single	41	31	41	9.137	0.166	35	29	49	14.437	**0.025**	4	14	95	12.034	0.061
	Married	183	67	118			166	99	103			36	60	272		
	Divorced	6	4	6			8	3	5			3	0	13		
	Widower	2	2	1			4	0	1			0	0	5		
Do you have children	No	50	37	52	13.332	**0.01**	43	35	61	17.559	**0.002**	6	19	114	6.028	0.197
	2 or less	88	32	69			91	44	54			18	32	139		
	3 or more	94	35	45			79	52	43			19	23	132		
Do you smoke	Yes	20	7	11	.682	0.711	17	6	15	2.561	0.278	2	4	32	1.322	0.516
	No	212	97	155			196	125	143			41	70	353		
Do you suffer from chronic diseases	Yes	65	28	49	.228	0.892	62	37	43	.160	0.923	11	17	114	1.518	0.468
	No	167	76	117			151	94	115			32	57	271		
Do you suffer from any non-chronic diseases	Yes	119	58	87	.580	0.748	116	61	87	2.594	0.273	20	30	214	6.331	**0.042**
	No	113	46	79			97	70	71			23	44	171		
BMI	Underweight	2	1	1	6.482	0.773	2	0	2	8.770	0.554	0	0	4	11.159	0.345
	Normal weight	40	24	38			41	26	35			8	13	81		
	Obesity I (Moderate)	57	28	39			58	32	34			11	17	96		
	Obesity II (Severe)	25	8	20			21	19	13			1	8	44		
	Obesity III (Very severe or morbid)	17	3	9			13	4	12			6	5	18		
	Overweight	91	40	59			78	50	62			17	31	142		

***lb:*** *low burnout;* ***md:*** *moderate burnout* and ***hb:*** *high burnout*

**Fig 2:**
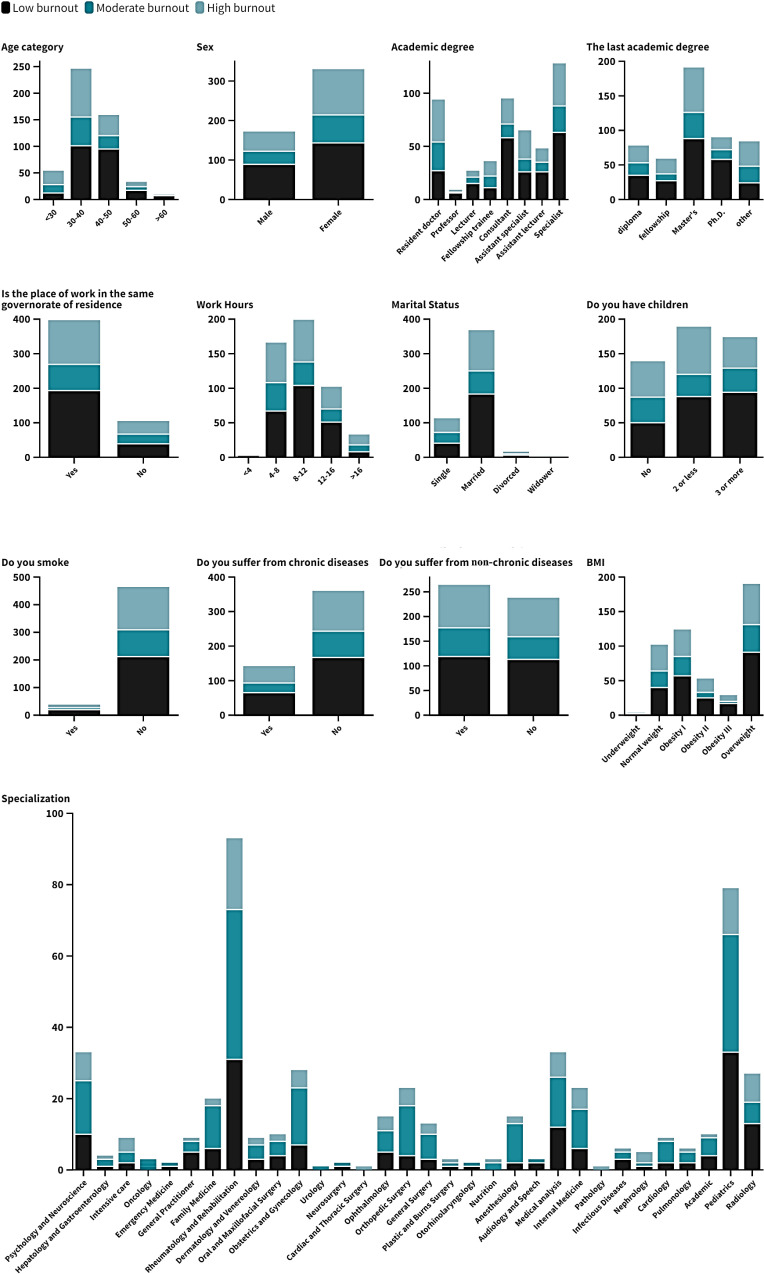
Stack column chart represent Personal Accomplishment through demographic structure.

**Fig 3:**
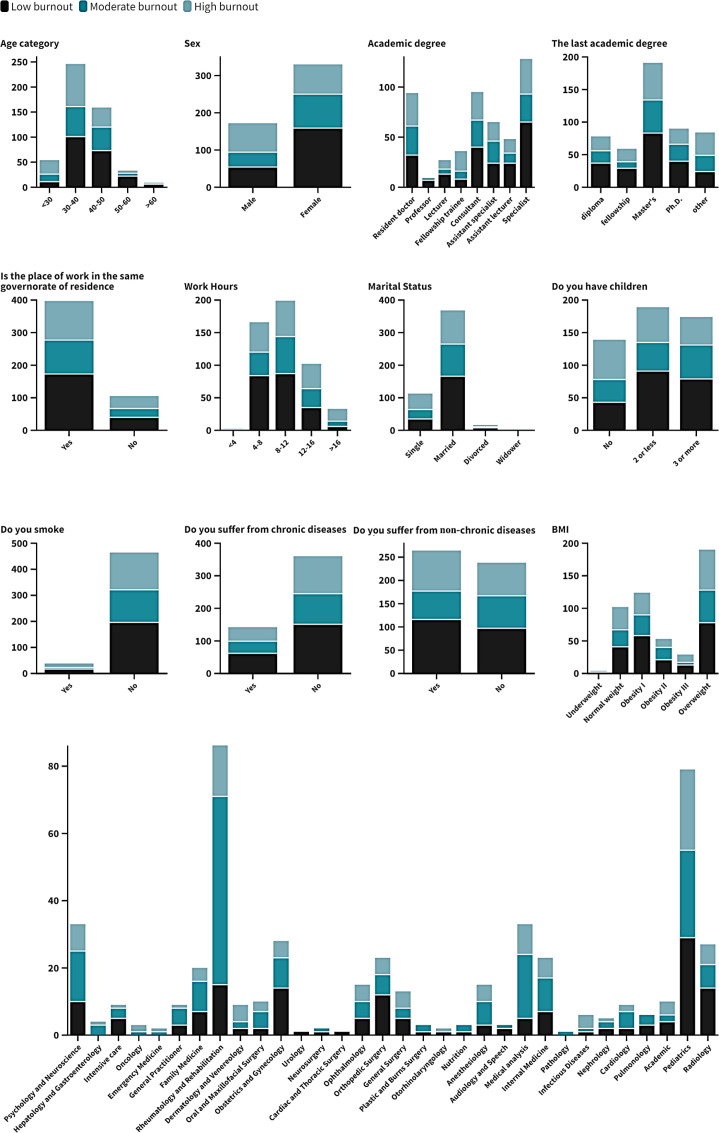
Stack column chart represent Depersonalization through demographic structure.

**Fig 4:**
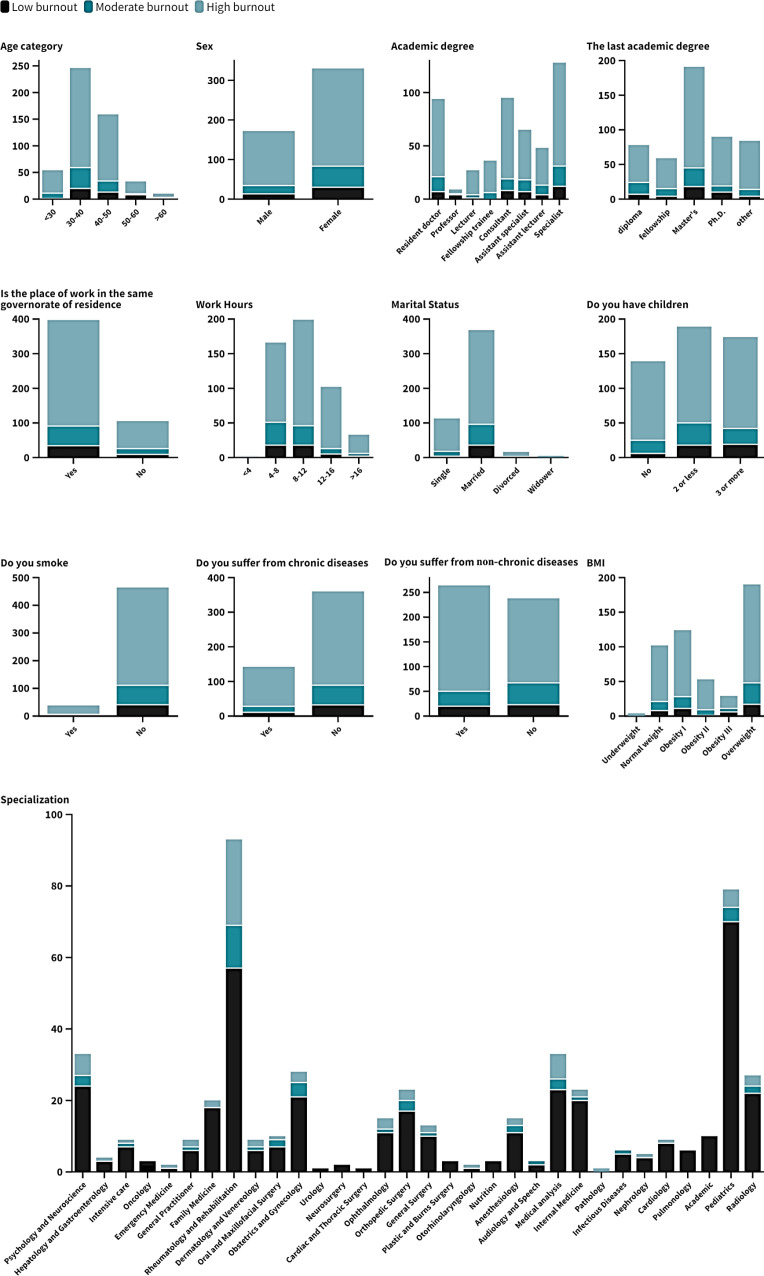
Stack column chart represent Emotional Exhaustion through demographic structure.

### Healthy lifestyle and personal control

**Fig 5:**
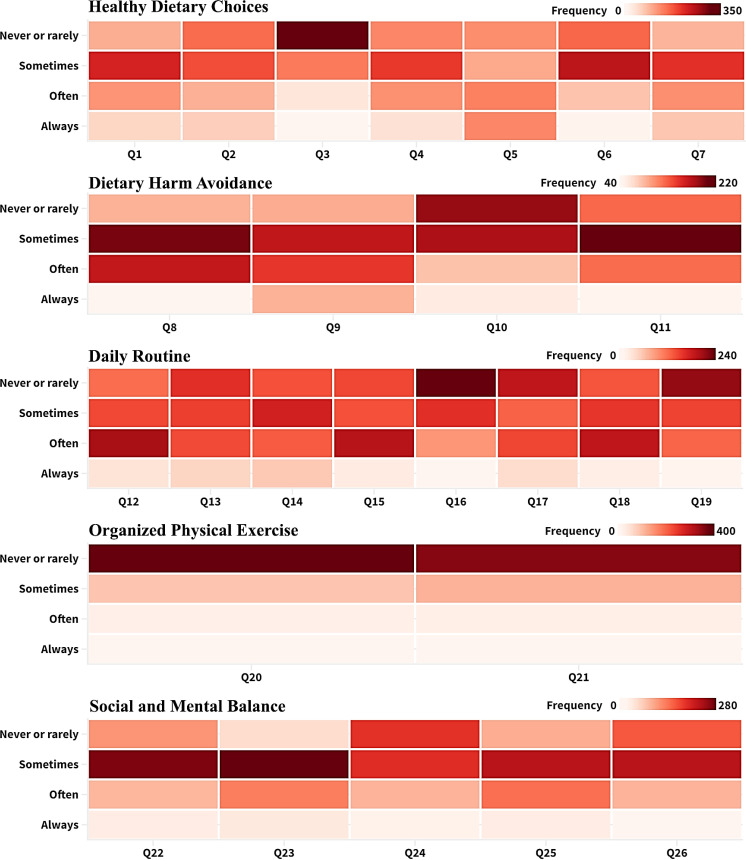
Heatmap represents the criterion of health lifestyle survey. (Color guide is provided above each figure).

Descriptive statistics were calculated for each variable in the dataset to characterize the answers of the participants. A 4-point Likert scale, from 1 (Never/Rarely) to 4 (Always), was used to evaluate how often individuals engaged in different health-related activities posed in the survey. There were reports of frequency distributions. (**Fig 5**).

With the use of principal component analysis (PCA) in PAST V4.12, the underlying dimensions of the lifestyle questions were determined. The data was checked to make sure it was suitable for factor analysis before the PCA was run. Bartlett’s Test of Sphericity showed that the correlation matrix was not an identity matrix and that there were sufficient linear correlations between the variables (χ^2^ (4005) =  21514.37, p 0.001). The sample size was sufficient for PCA, as measured by the Kaiser-Meyer-Olkin (KMO) criterion of sampling adequacy, which was 0.756.

**Fig 6:**
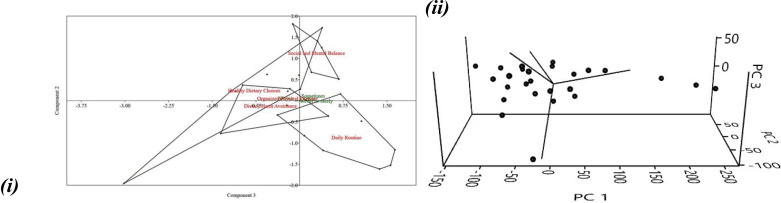
i & ii: PCA represent the (i) 2d and (ii) 3d to identify the underlying dimensions of the lifestyle questions.

After applying PCA, we found that 62.47 percent of the total variance could be explained by five components with eigenvalues greater than 1. The scree plot showed that there was a distinct split after the fifth component, confirming that these five should be kept. To improve their readability, the five components were rotated using a Varimax algorithm. Most variables loaded significantly on just one component in the rotated solution, indicating a straightforward structure (Fig 6 , i & ii).

Items with loadings higher than or equal to 0.3 were used to assign labels to the components. Labels included “Criteria 1: good dietary choices” and “Criteria 2: dietary harm avoidance,” “Criteria 3: daily routine,” “Criteria 4”: structured physical activity,” and “Criteria 5”: social and mental equilibrium.

Cronbach’s alpha coefficients were determined for the items loading on each component to evaluate the consistency of the criteria. All criteria were found to have strong internal consistency, with alpha values above the universally accepted 0.6 threshold ranging from 0.683 to 0.850.

Using the regression approach, factor scores were calculated for each participant in order to analyze the associations between the criteria and other variables in the dataset. Next, Pearson’s or Spearman’s correlation coefficients were used to find associations between the factor scores and demographic characteristics.

Criteria 1 (Good dietary choices) had a negative correlation with age, indicating that older people and those reporting lower levels of happiness tended to have fewer healthy eating habits. There may be a correlation between participants’ reports of lesser fatigue and their adherence to routines and schedules (Criteria 2: Dietary harm avoidance). The third criteria, “Daily Routine,” was positively correlated with both gender and professional status, suggesting that participants women had more regular daily routines. Participants who fulfilled criteria 4 (Organized physical activity) tended to have more orderly, predictable lives. With regards to Criterion 5, male participants were shown to be more cautious than female ones (Social and Mental Balance).

## Discussion

The present study investigated the risk factors or predictors that may cause physicians’ burnout syndrome by first indicating the demographic characteristics of the study participants that may had an impact on the level of burnout, Maslach Burnout Inventory (aMBI) which assesses three dimensions of burnout and finally the Healthy Lifestyle and Personal Control Questionnaire (HLPCQ), which measures the effectiveness of potential health-promoting treatments to improve people’s lifestyles and well-being.

Our study found that lower scores of personal accomplishments, higher scores of depersonalizations, and higher scores of emotional exhaustions were significantly observed among individuals aged 30-40 years. Similar to our results, a cross-sectional study was conducted among physicians working at Ankara Hospital, noticing that the 20-29 age group of young doctors had the highest levels of emotional burnout and depersonalization, and had the lowest personal success scores [14].

Another study was performed in Zagazig University Hospital with a selected sample representing the four different major specialties working in the hospital, supporting that emotional exhaustion and depersonalization were much greater in the 29-year-old age group than in the older age groups (more than 50 years). This can be explained by the fact that as people become older, they become more mature and more skilled in handling stressful situations without burning out [15].

Similarly, Christina Maslach “The American social psychologist” mentioned in a review article that Age is the demographic factor that has most consistently been linked to burnout among all those that have been researched. According to reports, younger workers experience more burnout than workers over the age of 30 or 40 as age and work experience are muddled, thus burnout seems to be a greater risk early in one’s career [16].

Regarding gender, we did not find significant differences, although a performed study in 2006 on general practitioners observed higher burnout levels among female physicians due to the conflict between professional and domestic tasks that female physicians are required to do [17]. However, the previous Croatian study we discussed before, found no significant differences regarding gender in burnout scores [14].

To our surprise, no significant observations were noticed regarding the levels of burnout in many different specialties of the study participants. However, another study observed a severely high level of emotional exhaustion and depersonalization among emergency department professionals compared to other specialties like paramedics. Many reasons make burnout more common among ED staff like nighttime work, job stress, and of course sleep issues [18].

The study was performed in Zagazig University Hospital and determined that different burnout levels are common between different specialties. They found that pediatricians had the highest mean scores for emotional exhaustion when compared to other specialties like internal medicine, general surgery, and gynecologists. A large percentage of pediatricians reported high emotional tiredness levels. On the other hand, only a small minority of them felt they had little personal accomplishment [15].

Even though, another study at Suez Canal University Hospital realized that hospital physicians had a considerably greater prevalence of burnout than family doctors. The highest prevalence was recorded among participants who work in the internal medicine division, followed by surgeons and emergency physicians. Pediatricians, on the other hand, had the lowest prevalence. Being married and working in a teaching hospital are both reliable indicators of burnout [19].

Another study confirmed the hypothesis that burnout scores may differ regarding the physician specialty, one performed in a Chinese cancer center found a high level of burnout among 21.1% of the oncology staff [20]. Meanwhile, the results among oncologists in the United States were nearly 45% of them experiencing symptoms of burnout and this is explained by the amount of hours per week spent working from home and concentrating on a particular type of cancer in clinical practice, both independently linked to the likelihood of burnout [21].

Concerning, marital status and the number of children, our study significantly observed lower scores of personal accomplishments among physicians with two or fewer children and higher scores of depersonalizations among married participants and individuals with two or fewer children. Although we did not find similar results regarding marital status from searching the literature review, a study among Croatian physicians observed no significant differences in burnout levels regarding marital status or even the number of children [22]. However, the study conducted at Zagazig University Hospital found that low scores of personal accomplishments were significantly higher among non-married when compared to married and this could apparently be due to the absence of the husband’s social support, which could act as a buffer against the consequences of stressful life events [15].

The burnout predictor we should pay attention to is the working hours, we significantly observed lower scores of personal accomplishments (high burnout) and higher scores of depersonalizations among participants working for an average of 8-12 hours per day. According to the World Health Organization (WHO), “Long working hours led to 745, 000 deaths from stroke and ischemic heart disease in 2016, a 29 percent increase since 2000, according to the latest estimates by the World Health Organization and the International Labour Organization” [23].

The study conducted among physicians working at Ankara Hospital confirmed that emotional burnout and depersonalization scores were higher among physicians who worked more than 8 hours [14].

Our study also examined the effects of residency, specialty, and master’s degrees on the levels of burnout to find lower scores of personal accomplishments between residents and those with master’s degrees and higher scores of depersonalizations among resident physicians and specialists.

Similarly, that study at Ankara Hospital confirmed that there were significant levels of emotional exhaustion with the greatest depersonalization levels among residents, and higher emotional burnout scores were significantly found among physicians who believed that the criteria of academic promotion were overpowering [14].

To our knowledge, this is the first study to investigate the lifestyle of Egyptian physicians by using the Healthy Lifestyle and Personal Control Questionnaire (HLPCQ) and resulting in five criteria. Our study presented the relationship between the physicians’ lifestyle or daily habits and other variables like their gender, age, and even their professional status.

It was observed that older participants and those who reported feeling less happy already have fewer healthy eating habits and that is criteria 1 “Good dietary choices”. The participants reported feeling less fatigued are adhered to routines and schedules and that is criterion 2 “Dietary harm avoidance”. The third criterion is “Daily routine” which showed a positive relationship between gender and professional standing, indicating that female participants had more consistent daily routines. Participants who fulfilled criteria 4 “Organized physical activity” tended to have more orderly, predictable lives. Regards criterion 5 “Social and Mental Balance”, it was found that male participants were more circumspect than female participants.

Other studies presented the lifestyle habits and well-being of the physicians in different patterns like the study performed in Bahrain. They found a clear pattern of unfavorable lifestyle habits and obesity among primary healthcare physicians. The authors reported percentages of the participants’ health conditions like hyperlipidemia, hypertension, BMI, and sleep time [24].

Another study performed in Pakistan presented the mental health well-being and work-life balance of the physicians using the Warwick Edinburg Mental Wellbeing Scale (WEMWBS) [25].

Another self-administered questionnaire was applied among primary health physicians in Saudi Arabia. The questionnaire included demographic information, medical history, physical activity, and stress levels. and food and smoking habits [26].

Our study recommends controlling burnout symptoms and searching for the factors that may contribute to improving physicians’ satisfaction with their careers. Also, in order to combat burnout, organizational leadership is crucial. Physician satisfaction and burnout are directly impacted by leadership traits, characteristics, and management approaches. The highest levels of physician satisfaction are produced by transformational traits and abilities like mentoring, coaching, establishing pride, talking about values and purpose, applauding achievements, and discovering unique needs and talents [27, 28].

It’s important to note that these are learnable abilities. Organizational leadership must show a commitment to fostering a culture of well-being, set an example for change, and address the issue of burnout at the systemic and organizational levels in order to achieve sustained and significant reductions in burnout [29].

The study by Safiye, et al., [30] explored the moderating role of resilience in the relationship between burnout and subjective well-being among medical workers in Serbia during the COVID-19 pandemic, finding that higher resilience levels significantly mitigate the adverse effects of burnout on well-being. Similarly, to our findings was also, the study by Safiye et al., [31] who examined burnout-related factors among healthcare professionals during the COVID-19 outbreak in Serbia, identifying high workload, emotional exhaustion, and inadequate support systems as key contributors to burnout. Both studies underscore the importance of resilience and targeted interventions in protecting the well-being of healthcare workers during crise.

### Study limitations

This study has some limitations that should be considered when interpreting the findings. First, the use of a cross-sectional design limits the ability to establish causal relationships between burnout and its associated factors. Second, the reliance on self-reported data may introduce response biases, including social desirability bias, which could affect the accuracy of the findings. Additionally, the study was conducted among Egyptian physicians, which may limit the generalizability of the results to other healthcare settings or regions. Finally, while efforts were made to ensure a diverse sample, the use of non-probability sampling techniques may have led to a sample that is not fully representative of the broader physician population.

## Conclusion

These findings highlight the intricate relationship between lifestyle factors and physician burnout. This study provides valuable insight into the correlation between the mental health of doctors and their physical health. Several outcomes are inversely correlated with harm avoidance, while burnout indicators are positively correlated with good eating, routine, social support, and exercise. These results point to the need for initiatives to improve the mental health of healthcare professionals and to establish initiatives to encourage healthy lifestyles. Medical personnel’ health can be improved by lifestyle adjustments.

## Abbreviations

COVID-19: Coronavirus Disease of 2019
EE: Emotional Exhaustion
DP: Depersonalization
PA: Personal Accomplishment
QoL: Quality of Life
aMBI: abbreviated Maslach Burnout Inventory
HLPCQ: Healthy Lifestyle and Personal Control Questionnaire
